# 以维奈克拉为基础的方案治疗急性混合表型白血病1例

**DOI:** 10.3760/cma.j.cn121090-20240108-00011

**Published:** 2024-11

**Authors:** 瑞华 米, 轶轩 马, 琳 陈, 琳 王, 佳 刘, 旭东 魏

**Affiliations:** 郑州大学附属肿瘤医院/河南省肿瘤医院，郑州 450008 The Affiliated Cancer Hospital of Zhengzhou University & Henan Cancer Hospital, Zhengzhou 450008, China

患者，女，59岁。2021年12月11日因体检发现白细胞偏低就诊于当地医院，血常规示：WBC 1.5×10^9^/L，HGB 105 g/L，PLT 258×10^9^/L，中性粒细胞计数 0.2×10^9^/L。骨髓细胞形态学显示原始细胞58％。染色体核型：47, XX, +8[2]/46, XX[5]。免疫分型倾向于急性髓系白血病（AML）。二代测序（NGS）检出ASXL1、DNMT3A、IDH1突变。当地医院诊断为AML。遂给予DA方案诱导缓解治疗，具体为：柔红霉素（DNR）60 mg/m^2^第1～3天，阿糖胞苷（Ara-C）100 mg/m^2^第1～7天，1个疗程后疗效评估示未缓解。后患者接受阿扎胞苷（AZA）联合CAG方案治疗，具体为：AZA100 mg第1～7天；G-CSF 300 µg，每12 h 1次，第0～14天；阿克拉霉素（Acla）20 mg/d第1～4天；Ara-C 15 mg，每12 h 1次，第1～14天，疗效评估示仍未缓解。后患者接受维奈克拉（Ven）单药治疗1个周期后获得缓解。后续分别接受了1个周期Ven、2个周期中剂量Ara-C、1个周期Ven巩固治疗（具体用药不详）。在Ven维持治疗中疾病复发。融合基因检测示BCR::ABL1（p210）13.97％。染色体核型：46, XX,t（9;22）（q34;q11）[4]/46,XX[10]。

患者于2023年2月21日入住我院。入院后进行形态学、免疫学、细胞遗传学、分子生物学检查。骨髓象：增生明显活跃，原始幼稚淋巴细胞占42.0％，原始幼稚单核细胞占8.4％；流式细胞术免疫分型：异常髓系原始细胞占12.67％（表达CD34、CD117、HLA-DR、CD38、CD13、CD81，弱表达CD33、cMPO），异常幼稚B淋巴细胞占34.78％（表达HLA-DR、CD38、CD19、CD10、CD22、cCD79a、CD81，部分表达CD34、CD20）；染色体核型：46,XX,t（9;22）（q34;q11）[6]/46，XX[4]；融合基因检测示：BCR::ABL1/ABL1（p210）（IS）68.79％；基因突变检测示：IDH1突变阳性，后修改诊断为B/髓系急性混合表型白血病（MPAL）伴BCR::ABL1。后给予VP（长春新碱2 mg第1、8天，泼尼松60 mg第1～14天）联合伊马替尼（400 mg每日1次）、Ven［100 mg第1天、200 mg第2天、400 mg第3～20天，100 mg第21天始（因合并肺部真菌感染联合唑类药物）］方案治疗。治疗第16天复查骨髓细胞形态学显示原始细胞占4.0％，BCR::ABL1/ABL1（p210）6.28％，流式细胞术检测微小残留病（MRD）示异常幼稚细胞占0.88％。治疗第28天复查骨髓细胞形态学未见淋系原始细胞，髓系原始细胞占10％。MRD检测显示异常髓系原始细胞占3.15％。

因患者一般情况较差，期间肺部感染较重，不适合继续接受标准方案治疗，遂给予Ven（100 mg第1～28天，持续联合伏立康唑抗感染）联合氟马替尼（600 mg每日1次）治疗。后多次复查骨髓示完全缓解，流式细胞术检测MRD示阴性。BCR::ABL1/ABL1（p210）（IS）波动于0.0012％～0.0026％，患者因多种原因拒绝异基因造血干细胞移植，Ven联合氟马替尼治疗期间耐受性良好。

2024年3月28日因院外持续发热7 d，再次返院，入院后查血常规示：WBC 0.68×10^9^/L，HGB 84 g/L，PLT 122×10^9^/L，中性粒细胞计数0.11×10^9^/L；骨髓象：增生活跃，原始细胞占90.6％，流式细胞术检测MRD示：共获取有核细胞约500 000个，其中CD34^-^CD117^+^CD13^+^CD33^+^HLA-DR^dim+^ CD38^+^CD19^+^CD10^-^CD20^-^CD22^-^CD81^+^CD45^dim^异常原始细胞占27.08％，染色体核型：46,XX, inv（3）（p21q26）, t（7;15）（p21;q23）[6]/46,XX,del（7）（q31q35）[2]/46,XX[2]。二代测序示：IDH1，KRAS（+），BCR::ABL1/ABL1（p210）（IS）（−）。患者疾病再次复发，且发生克隆演变，因再次新型冠状病毒感染致重症肺炎于2024年4月8日死于呼吸衰竭。

